# Sovereignty, privacy, and ethics in blockchain-based identity management systems

**DOI:** 10.1007/s10676-020-09563-x

**Published:** 2020-11-30

**Authors:** Georgy Ishmaev

**Affiliations:** Technical University of Delft, Van Mourik Broekmanweg, 2628 XE Delft, The Netherlands

**Keywords:** Identity management, SSI, Sovereignty, Privacy, Blockchain

## Abstract

Self-sovereign identity (SSI) solutions implemented on the basis of blockchain technology are seen as alternatives to existing digital identification systems, or even as a foundation of standards for the new global infrastructures for identity management systems. It is argued that ‘self-sovereignty' in this context can be understood as the concept of individual control over identity relevant private data, capacity to choose where such data is stored, and the ability to provide it to those who need to validate it. It is also argued that while it might be appealing to operationalise the concept of ‘self-sovereignty’ in a narrow technical sense, depreciation of moral semantics obscures key challenges and long-term repercussions. Closer attention to the normative substance of the ‘sovereignty’ concept helps to highlight a range of ethical issues pertaining to the changing nature of human identity in the context of ubiquitous private data collection.

## Introduction

Any technical solution dealing with the issues of human identity management and private data is intertwined with ethical challenges. This is especially so in the case of Self-Sovereign Identity (SSI) solutions enabled by the developments in blockchain technologies. To an extent, these systems—like cryptocurrencies—are also influenced by the ambitions to push towards the decentralisation of trust in complex systems and reduce reliance on trusted third parties. Proponents of these solutions argue that SSI systems can bring enhanced privacy, data security and full controls over their digital identities to individuals (Tobin and Reed 2016; Allen 2017; Ma et al. 2018; Wagner et al. 2018). These claims are loaded with ethical assumptions seemingly targeting the very core set of concerns regarding privacy and identity in the emerging socio-technical structures of contemporary society. And as with many other similar claims, it is hard to disentangle actual technological implementations from promises, unsupported assumptions, and even misinterpretations, constituting the all too familiar retinue of blockchain technology applications.

The task to qualify these claims becomes even more complicated once we consider that SSI systems, like many other blockchain implementations, are still in the experimental stages of development. However, what stands them apart is that these experiments seem to deal with hypersensitive issues of individual identity and identification. And as Sen (2007) vividly demonstrates on the historical lessons from the twentieth century, experiments on identity can have dramatic and undesirable consequences. It is also evident that a proper moral evaluation of any technical implementations cannot be carried out in the vein of a naive technological determinism. The complexity of technology development cycles, does not always guarantee that even the noblest moral aspirations of its creators will directly translate into desirable social outcomes. With the wider adoption, technologies become embedded into preexisting social, economic and political contexts and resulting socio-technical phenomena not only surpass the ambitions of their creators, but sometimes also bring outcomes completely opposite to the intended.

This is especially true for blockchain technologies: those key properties—malleability, low costs for entry, and potential for rapid adoption on a scale—make accurate predictions very problematic (Filippi and Hassan 2018). This is even more so when such predictions involve reflections on such philosophically loaded phenomena as ‘identity’ and ‘sovereignty’. Unsurprisingly then, some critics in the field of SSI solutions developments are calling for the heavy baggage of philosophical reflections—which only distract the developers from bringing the practical benefits of technology for society—to be abandoned. (Ma et al. 2018). And arguing that concepts of ‘identity’ and ‘sovereignty’ in SSI solutions should be treated as technical concepts detached from the semantic genealogy of these terms (Wagner et al. 2018).

To understand why such semantic isolation might be problematic we need to consider the problem commonly occurring in the interdisciplinary contexts—conceptual slippage. At a high level of abstraction the identification of entities in the technical system is a general problem in computer networks. Depending on the context we may want to identify elements in the Internet of Things architecture, organisations or simply network endpoints. In that sense verification of identities and credentials is a broad technical problem. Identity management solutions, however, can also be used to share identities and credentials for natural persons. The risk of conceptual slippage here means that the concept originally borrowed from humanities and reduced to a narrow meaning in computer science, yet again gets translated into different context without taking into account different level of abstraction (Ishmaev 2018). In a worst case scenario, reducing human users to mere endpoints in the model of a system for the sake of convenience.

The problem with this approach is that even if such a conceptual model explicitly abstracts away from the moral-philosophical considerations it does not mean that the resulting solution will be morally neutral. As Manders-Huits (2010) argues, any human identity management system inevitably carries a special set of moral concerns. Primary of which is a nominalisation of identity—the reduction of personal identity to a set of forensic descriptions; a process that ignores fundamental moral considerations of respect for persons. The complication also comes from the fact that even without direct linkage to individuals, such identity relevant private data can structure interactions with them in ways that invoke moral concerns (Manders-Huits and van den Hoven 2008). Similarly, Shoemaker (2010) argues that it is not possible to disentangle moral aspects of private data management from the issues of self-determination and identity, given the changing nature of identity formation in the digitalised world defined by the ubiquitous collection of private data. It can be argued thus, that the task to explicate key moral concerns driving the development of SSI technologies is hardly an optional exercise.

This paper aims to outline the context of the social and technological developments that define the moral concerns motivating the development of SSI technologies. Such an investigation is impossible without locating the common normative theoretical and technological roots of SSI systems and other blockchain implementations. Not only is this necessary to clear up some basic misconceptions, but also to understand the rather special status of moral concerns surrounding the very idea of ‘self-sovereignty’ in a broader context of blockchain technologies. From its very first instantiation, blockchain technology—presented to the world as the Bitcoin application—has been deeply intertwined with the issues of individual freedoms and rights in the world defined by information-communication technologies. And while from the technical perspective blockchain itself is not a necessary element for SSI (though this is possibly the most feasible approach at the moment), the concept of ‘self-sovereignty’ in both of these solutions is historically rooted in the tradition of cypherpunk thinking (Hughes 1993). A quote attributed to Bitcoin’s anonymous creator, Satoshi Nakamoto, explaining some motivations behind the project, is worth citing here: “we can win a major battle in the arms race and gain a new territory of freedom for several years.”^1^

This statement, which may seem like a colourful metaphor at a first glance, refers to the set of moral concerns regarding issues of autonomy, self-determination and individual rights in the context of changing social structures that are more and more defined by the new technologies. In that sense the “arms race” refers to the fact that with the growing dependence of a contemporary society on communication infrastructures, the adversarial thinking initially constrained to the fields of cybersecurity and cryptanalysis, spilled over into other contexts of social relations on an unprecedented scale. In fact, this apprehension was highlighted much earlier by David Chaum (1985). He argued that a society dependent on computer networks in all aspects of everyday life, risks extending the logic of computer security into many other realms of social relations. This, in turn, opens up a Pandora’s box of a dossier society, repeating rigid hierarchical structures of centrally controlled communication systems, built around mandatory identification, mandatory trust assessment, and scrupulous record keeping of past behaviour for individuals. It can be said that Bitcoin emerged from this line of thinking; as an attempt to change the balance of power between entities racing to control key communication infrastructures, and individuals becoming more and more dependent of those infrastructures.^2^

Unsurprisingly then, the idea of self-sovereignty, not only in respect to financial sovereignty of cryptocurrency solutions, but in a broader politico-philosophical context takes prominent place in different implementations of blockchain protocols (Reijers et al. 2016). I argue, however, that the normative concept of ‘self-sovereignty’ should be distinguished from the technical term of ‘self-sovereign identity’ used as a label for a rather broad family of technological standards and solutions. And as this paper aims to demonstrate, the gap between moral-philosophical and technical meaning of these concepts should not be ignored, as the moral desirability of SSI implementations directly depends upon our capacity to bridge this gap. I argue that the normative ideas of ‘self-sovereignty’ can be better understood through the prism of *sovereign powers*, outlined by Locke in his critique on the moral sources of authority in a society (Locke et al. 2003 [1823]).

“New domains of sovereignty” section of this paper looks into the moral issues caused by the asymmetric distributions of powers in the technological infrastructures for identity management, interpreted as the manifestations of competitions for *functional sovereignty*. “Technical components of SSI systems” section takes a high-level overview of the key technological components for SSI systems, in order to locate the moral significance of these systems in the broader socio-technical contexts. “Moral foundations of sovereign rights” section provides a moral-theoretical grounding of the idea of self-sovereignty combining insights from the Lockean classic liberal critique on individual rights and the more recent philosophical tradition of thinking on the moral foundations of informational privacy. The paper concludes with arguments on the appreciation of ethical risks of identity nominalisation in SSI systems.

## New domains of sovereignty

The concept of *sovereignty* has a long history and a variety of meanings in different discourses. Thus, for the first step of our investigation it is crucial to outline the unique role that the concept of sovereignty enjoys. One such peculiar aspect of sovereignty is highlighted by Kalmo and Skinner (2010), who argued that the ambiguity of sovereignty has certain historical depth, providing a reflection of past efforts to give it content, rather than the result of a conceptual confusion. As such, most of the time arguments about sovereignty are not merely scholarly debates on the meaning of terms, but rather arguments about allocation of power. Similarly, Werner and De Wilde (2001) providing analysis of the concept of sovereignty in the context of international law point out that treating sovereignty as a purely normative concept is equally as erroneous as trying to define it as a purely descriptive one. First and foremost, sovereignty is a claim—not merely a factual claim or merely a normative one, but also a *legitimising* claim. What is meant by this, is that a successful claim to sovereignty aims to establish a link between a certain institutional fact and certain rights and duties following from this fact. Thus, it can be said that the unique liminal status of the concept of sovereignty means that the attempts to ascribe sovereignty reflect a struggle over whom or what institution ought to posses it. It is never merely a description of empirical fact, but also an attempt to legitimise and justify a certain state of affairs.

To understand some empirical aspects of these transformations in the context of our investigation, it might be helpful to consider an even more peculiar concept of a *functional sovereignty*. This concept was first introduced by Riphagen (1975) in the context of international maritime law to describe a new phenomenon of legal rights occurring outside of the scope of territorial rights traditionally defined and circumscribed by the context of national sovereignty. He suggested an application of a concept of functional sovereignty in those cases where there is said to be a stateless domain, yet where there seem to be some government in the absence of territory. It can be said that new information and communication technology (ICT) infrastructures, brought about by the creation of the Internet and other technological developments, effectively create new domains outside the scope of traditional territorial divisions. This is, of course, a multifaceted issue, covering numerous phenomena such as, for instance, ‘Balkanization of the Internet—’ attempts by state actors to translate national boundaries into virtual spaces,^3^ or the more recent generation of ‘encryption wars—’ manifesting as a struggle between various corporate and state actors to control vast amounts of personal data.^4^ And interestingly enough, sometimes this struggle for functional sovereignty even spills over into, and overlaps with, the traditional domains of territorial sovereignty, with the developments of smart-city infrastructures.^5^

The emerging domain of new identity management systems, also unsurprisingly became an arena of this contest. The key defining trend here is the shift towards the monopolistic control of technological platforms, based on the high capital concentration, creation of dependencies for its users, and control of market flows through the occupation of structural position (Rahman and Thelen 2019). The latter point has particular salience in the context of human identity management systems. Technological giants such as Facebook and Alphabet aggressively try to inject themselves into all kinds of identity based online transactions providing end-user identity solutions, built on top of their massive private data silos.^6^ Combining private data from its various surveillance platforms, they manage to aggregate incredibly fine-grained and full profiles both online and offline on a truly staggering scale (Schmidt 2018; Holmes 2020). Furthermore, even though these two companies are exemplars of self-proclaimed sovereigns striving to control new identity domains, they in fact represent just a tip of an iceberg, which is largely an opaque, global private data industry that includes corporate and state actors of various calibers and ambitions, trying to aggregate dossiers, consumer profiles and ultimately silos of identities (Ramirez et al. 2014; Christl and Spiekermann 2016).

And in line with Werner and De Wilde (2001) observations on the legitimising aspect of sovereignty claims, such companies also engage in attempts to legitimise their privileged structural positions on the pseudo-moral grounds. As chief privacy officer of Facebook, Chris Kelly, expressed these claims: “We’ve been able to build what we think is a safer, more trusted version of the Internet by holding people to the consequences of their actions and requiring them to use their real identity” (Kirkpatrick 2011). Furthermore, these attempts to exercise such self-legitimising functional sovereignty from the position of ‘brute facts’ went unchallenged by regulators and the public up until more recent revelations around large-scale data abuses that came to light in the context of ‘Cambridge Analytica’ scandal (Adams 2018).

These developments in identity management systems, however, are not limited to commercial companies. Some further insights on the key trends taking place on the global scale provide two ambitious nation scale projects: ‘Aadhaar’ in India, and the ‘Citizen Social Score’ in China. These projects can be seen as attempts by the respective national governments to extend the scope of national sovereignty in the new domains enabled by the developments in the technological surveillance apparatus. And as with other claims for sovereign power, these are deeply entangled with moral claims aimed to legitimise these new extended powers. Aadhaar—a centralised identity database for Indian citizens built around biometric identification—presents an interesting example of what Kalmo (2010) characterised as a paradox of sovereignty and illegality. Dixon (2017), providing a timeline for the implementation of Aadhaar, points out that the system was effectively put in place in the absence of any connected regulatory and policy guidance.^7^ At the same time, the introduction of this system was justified to the general public, largely on moral grounds, as a necessary implementation capable of preventing fraud in the distribution of state subsides.

And just like in the case of corporate identity management systems, moral argumentation aimed at justifying Aadhaar implementation is overshadowed by numerous data breaches, instances of data abuse, and function creep that effectively undermine the validity of this justification (Dixon 2017).^8^ But, an even more fascinating example of this trend in the development of identity management systems is presented by an ambitious project by the Chinese government: ‘Social Credit System’ (SCS). This system, envisioned as an integrated registry of Chinese citizens, is maintained on the basis of collaboration between various state agencies and commercial companies. But unlike other state identity management systems, SCS goes beyond mere forensic purposes and implements an explicit system of scores for profiled citizens designed to reflect their ‘trustworthiness’. Furthermore, having a low or high score has very real material consequences for profiled individuals, formalised in the system of respective rewards and punishments (Ohlberg et al. 2017; Engelmann et al. 2019).

In that sense SCS presents an ‘endgame’ example of a complete system for surveillance and profiling, which in itself carries profound moral issues. However, it also serves as a fascinating example of an effort of the Chinese government to exercise its sovereign power in a completely new domain. In itself the claim for the sovereign right to define moral identities of its citizens is not new for Chinese or many other governments. It can be traced back in history probably as far back as the theocratic societies of the Bronze Age as a ‘controlled mechanisms of identity formation’ through moral codes of conduct (Koskenniemi 2010). What makes SCS implementation historically unique, however, is firstly its scale, and secondly, the modality of its normative components. It is not merely a system prescribing moral norms and identities, but effectively an integrated control apparatus that ensures adherence to prescribed norms through automated rewards and punishments. Thus, it is not merely a ‘moral code of conduct’, but a grand socio-technical engineering project aimed to eliminate, ‘untrustworthy elements’ and ‘black sheep’ in society (Ohlberg et al. 2017). Unsurprisingly, the system carries a distinct totalising and controlling character, prioritising and emphasising behaviour that can result in a lower scores and consequent punishments (Engelmann et al. 2019).

SCS thus, represents a highly peculiar (and disturbing) illustration of a trend characterised by Koskenniemi (2010) as a transformation of sovereignty from limiting sovereign powers, to enabling powers. As such, sovereign power is not only state power used to limit certain actions of its subjects, but rather power to define the very category of a subject. This is also a moral problem characterised by Sen (2007) as a denial of choice and responsibility for one’s own identity, when individuals are prescribed with ‘true’ singular identities, stemming either from national or religious identification. And this shift also largely defines manifestations of functional sovereignty in the domains of identity management systems, not being limited to state entities but also found in actions and strategies of hybrid and corporate actors. From that perspective, a widely cited statement by Facebook’s founder Mark Zuckerberg takes on a new meaning: “Having two identities for yourself is an example of a lack of integrity” (Kirkpatrick 2011). Indeed, this statement is not merely an opinion, or expression of a moral view by a private person, but effectively a claim by a transnational identity platform to define criteria for ‘good identity’ as a sovereign power.^9^ Similar claims can also be found in the attempts to establish epistemic authority of technological solutions for the ‘personality assessment’, claiming the ability to reveal one’s ‘true’, ‘real’ identity (Youyou et al. 2015).

The key issue here is that a singular identity persistent across the range of contexts, is not always morally desirable, and not all context of identification require such identity (Shoemaker 2010). Many pseudonymous or anonymous transactions involve presentations of simple credentials and minimal sharing of private data. However, once any transaction involves the presentation of ‘identity’ provided by an intermediary, rather than a disposable ‘persona’ or even simply anonymous credentials, this not only injects Facebook-like intermediaries into any such transaction, but also gives them extraordinary gatekeeping power. And this trend manifests itself particularly vividly in the workings of the data-brokers’ industry and various credit rating agencies, providing all types of identity assessments for financial institutions, marketers and employers (Ramirez et al. 2014). In a sense, these developments also characterise an identification creep, where types of social relations that do not require persistent identification of counteracting parties become supplanted by epistemically asymmetric identity based relations, driven by the logic of adversarial thinking. This thinking defines the relations where each party tries to find as much as possible about their counteragents to achieve an information asymmetry to ones’ own advantage in a manner of a zero-sum game.^10^ These systems, implemented on the basis of proprietary algorithms, create truly Kafkaesque, scenarios where completely arbitrary entities wield power to define the criteria for ‘good’ or ‘bad’ identities, not justified by legislation or any kinds of social agreements, but merely as brute facts (Lecher 2019).

Furthermore, such profiling and rating systems not being confined to legal vacuum spaces, partially become adopted and legalised post-factum; once again reinstating the paradoxical nature of sovereignty as a capacity to convert extreme cases of illegality into new standards of legality (Christin et al. 2015). From this investigative empirical perspective, moral claims supporting implementations of these systems are first and foremost legitimising claims. Any attempts to establish a link between certain institutional fact and certain rights and duties following from this fact, become successful when new standards of legality are established (Werner and De Wilde 2001). These observations not only suggest another dimension to the problem of the ‘arms race’ and ‘territory of freedom’ highlighted by Satoshi Nakamoto, but also define a very complex background for the implementation of SSI systems.

As we can observe, this fixation on identity, warned against by Sen (2007), is not going away in the twenty-first century. On the contrary, fascination with the identity, the framing of identity as ‘the solution’ to grand ethical challenges, drives the development of identity experiments of an unprecedented scale, and of unprecedented ambitions. And quite often SSI solutions are seemingly surrounded by the same grandiose ethical claims as Aadhaar and Social Credit Score systems. Indeed, among its ambitions, the transnational alliance ID2020 claims to offer solutions to such global issues as economic inclusion in developing countries, humanitarian refugee crises, world hunger and many others.^11^ This highlights a certain paradox that state actors and transnational corporations—all those entities that can hardly be suspected as being champions of techno-libertarianism and libertarian interpretation of individual rights—seem to embrace the ‘individual empowerment’ and other aspirations of SSIs, and advocate for urgent adoption of new identity solutions. An unflattering parallel with other experimental identity management systems causes a valid apprehension that these claims may also fall into a category of legitimising claims, devised merely to justify and validate instalments of new socio-technical structures preserving some forms of intermediary privileges. To address these concern we need to look into the key technical components that could be considered definitional elements of SSI solutions.

## Technical components of SSI systems

In comparison with the original blockchain implementations, it is difficult to highlight one single project that could be representative of SSI technology in the same sense as Bitcoin is a flagship example of blockchain-based cryptocurrencies, or Ethereum is a prototypical protocol for smart contracts. At the moment there are at least a hundred different projects that employ blockchain technology in order to provide functionality of digital identity in one form or another.^12^ All of these projects are in different stages of development, some of them lacking sufficient documentation that would allow for closer scrutiny. And considering how generally volatile the field of blockchain-based projects is, it is reasonable to highlight those in the later stages of development that go beyond mere proof-of-concept implementations. A couple of these projects are: the ‘uPort’ identity project developed by ConsenSys,^13^ and the Sovrin project by Evernym.^14^ At this point it is difficult to predict whether any of these solutions will be widely adopted, so it does not seem feasible to go into details of their particular implementation. But it is helpful to get a high level overview of the underlying technology that highlights the basic properties of SSI implementations present in most of these projects to a certain degree.

Considering that any SSI at this point is very much a bleeding-edge technology, there are no clearly established standards. However, impressive work in this area has been accomplished by W3C Credentials Community Group.Three specific technical components that comprise and enable the idea of SSI technology, present key interest here.^15^ The starting point here is to consider that public/private key encryption underlying most of the online interactions (such as messaging) can also be used to establish identities of the interacting parties. The method of two-key encryption (or asymmetric cryptography) can be used both to encrypt messages and sign them. For instance owner of key pair (public and private key) Alice publishes her public key, so that Bob or anybody else can use it to encrypt message in such a way that only Alice can decrypt it using private key. Or alternatively, Alice can sign a message with her private key, so that Bob using the public key can verify that the message was indeed signed by her (given that Alice is a unique holder of the private key). This can be done with the help of a Public Key Infrastructure (PKI) which enables the exchange of keys between the parties and links names to the specific keys. Traditional PKIs are managed by the centralised trusted parties, such as certificate authorities or messaging service providers. The first crucial concept in the SSI schema is the Decentralised Public Key Infrastructure (DPKI)—essentially a data base containing public keys. The main novelty of DPKI is that, using blockchain as a decentralised database, it can radically reduce reliance on trusted parties while at the same time ensuring security from manipulation, censorship, or compromise (Allen et al. 2015).

With the help of DPKI, identity owners can register their decentralised identities associated with public keys on the blockchain without dependance on any centralised registrars (thus “self-sovereignty”). Schematically it can be said that DPKI forms the base layer allowing for another key component of SSI system—decentralised identifier (DID). Defined as a technical standard, in its idea DID is similar to a Uniformed Resource Identifier. DID, however, points to entities (endpoints associated with natural persons or organisations for instance) rather than Web resources. And unlike a URI, the DID Document typically contains cryptographic material that enables authentication of the entity identified by DID. In itself, generic DID contains an identifying string of symbols as an ID index and metadata, together called the DID document—a machine readable structured piece of data—and metadata called the DID document. In its most basic form, this identification scheme can include ID strings as a designation of the owner, information about the context of identification, cryptographic methods of authentication (specific public keys), and pointers to the method of authentication (specific blockchain).^16^

Such identities in themselves provide limited functionality of course. The third crucial concept of SSI, however, makes a significant difference: the capacity to issue verifiable credentials. From the user’s point of view, a verifiable credential is a digital, cryptographically signed document containing certain claim(s) about its holder—such as being a of certain age or being licensed to operate a vehicle—essentially similar to physical credentials. Practically, verified credential implementation proposed by W3C uses DIDs as subjects of claims and DID documents as root records for digital identities. This scheme allows individuals to exchange credentials in a privacy-preserving manner. An individual can potentially generate multiple DIDs for interactions with different parties, choose different parties to sign his/her verifiable credentials, and present only specific verified claims (such as age) to minimise private data disclosures.

Thus, it can be said that self-sovereignty here is the concept of individual control over identity relevant private data. Primarily the capacity to choose where such private data is stored, and the ability to provide it to those who need to validate it. To illustrate it in a simplified way, this scheme allows for a secure connection between peers to communicate securely and share credentials. Bob can choose to share his age-related information with a vendor to buy alcohol, and provide a credential with an attribute ‘over 21’ signed by a trusted issuer. The vendor, in turn can verify that the owner of the credential is over 21, and can verify that the credential was given to the person who possesses the private DID that has been shared with the vendor. Bob can also generate any number of DIDs, one for each digital relationship (i.e. one per vendor in this example) and share unique proofs of the over 21 credential with each vendor. This prevents Bob’s actions from being correlatable across the vendors.

This scheme is more complicated in practice and can employ additional cryptographic tools such as zero-knowledge proofs. Using this method for extra obfuscation, Bob can prove to a vendor possession of a valid signature without revealing the signature itself (Smith and Khovratovich 2016; Stokkink and Pouwelse 2018). Such obfuscation of private data is a very promising approach to enhance the privacy of individuals who might use such solutions. Still, the key novel element of this approach is arguably enclosed in the decentralisation properties of SSI schemes. It is suggested that in the future blockchain-based DPKIs will have many cross-references to verified credentials forming a cross web of trust, that it will be possible to issue credentials without reliance on trusted authorities such as a motor vehicle authority, etc. (Tobin and Reed 2016).

The technical implementation of the uPort project in a certain sense is closer to existing cryptocurrency blockchains since it is built on the basis of the Ethereum public blockchain. Ethereum, however, is not a cryptocurrency specific chain, since it can be seen as a distributed computation protocol capable of storing and executing programs called ‘smart contracts’ on virtual machines. Using Ethereum-specific protocols, the uPort SSI scheme creates a number of layers for the management of digital identities and verifiable claims. This scheme is different in some respects from the generic one given above. DIDs in uPort are implemented as smart contracts, where the blockchain address of a smart contract serves as a persistent identifier. DID document functionality in uPort is split between Controller Contract, Proxy Contract and Application Contract.^17^ This scheme makes uPort a public infrastructure on the basis of blockchain layer. Using a smartphone app, any user can issue and mange credentials on uPort, and connect these credential to private data stored off-chain (in any other data base separate from Ethereum blockchain).

The Sovrin project takes a different approach, aiming to create full infrastructure for the implementation of SSI from scratch. As such Sovrin runs its own blockchain, which employs specific architecture and original consensus protocol. Sovrin blockchain is branded by its developers as a ‘public-permissioned’ ledger, as opposed to a public blockchain. This means that while any entity can use this scheme to manage credentials, in order to become a node in the basis layer network, an entity has to be vetted by the Sovrin foundation (which is an incorporated entity). Furthermore, only a limited number of all nodes have the right to add new records to the blockchain database, thus making this blockchain essentially private. According to Sovrin, decentralisation in such a network can be achieved via economic and political independence of nodes distributed in different countries, complemented with legally binding agreement for nodes formalised as ‘Sovrin Trust Framework Agreement’. Individuals who wish to use Sovrin identity management for personal purposes are also supposed to sign a legally binding agreement. It can be argued thus, compared to public blockchains Sovrin aims for a different approach to the decentralisation of DPKI. The distinction here is the decentralisation of governance vs. the decentralisation of infrastructure. Whereas the Sovrin infrastructure is no longer centrally located geographically (as happens with Bitcoin mining pools for instance), the governance—which is the important part in open permissionless systems—is still centralised or federated rather than decentralised.

What can be derived from these technical descriptions is that SSI solutions in their current implementation are first and foremost tools for the management of private data. *Sovereignty* here is largely interpreted as an ability to share verified credentials in a way preferring minimal data disclosures. Accordingly, *Self*-*sovereignty* is understood as the concept of individual control over identity relevant private data, capacity to choose where such data is stored, and the ability to provide it to those who need to validate it, without relying on any centralised repositories of identity data. Furthermore, while it can be said that these solutions are enabled by blockchain technology, at the fundamental level of general design assumptions SSI systems fall into a completely different category than Bitcoin. The key conceptual difference at a high level of abstraction stems from basic assumptions laid in the Proof of Work (POW) consensus protocol in the foundation of Bitcoin blockchain (Narayanan et al. 2016). This approach demands the contribution of computationally expensive resources from network participants on a competitive basis to achieve network security, and thus abolishes the traditional requirements of identity, trust, and permissions. SSI solutions, on the other hand, aim to provide the identity layer on top of the blockchain protocol, in the traditional paradigm of identity-based systems.

Thus, it can be said that sovereignty for individuals takes significantly different forms in SSI solutions and cryptocurrencies. Bitcoin public network absolving requirement of identities both from those who would like to use or contribute resources, it does at least—hypothetically—aim for egalitarian decentralisation.^18^ The concept of a trust network lying in the foundation of SSI presupposes, of course, that any entity can become verifier, but there are fundamental differences between levels of trust in these entities in the real world. Verifiable credential signed by a state entity or transnational bank carries a much higher trust value, both in the scope and in the weigh of claim validity (Wagner et al. 2018). Thus, entities possessing certain ‘trust capital’ in the socio-economic sense, quite justifiably expect to claim advantageous positions in the future SSI infrastructures, serving as nodes of trust. This, of course, is not morally problematic in itself, but rather highlights the practical limits of sovereignty that individual users may hope to exercise, in contrast with aspirational libertarian ideal of self-sovereign authority (Loffreto 2012). Alice can carry SSI based credential with her in the chosen storage and use it where she wants. However, it is also not up for Alice to decide what type of credentials she has to present when this transaction occurs in the existing scheme of power relations.^19^

From that distinction it can be observed that the term SSI itself has two distinct meanings, in the normative sense of the ‘Self-sovereignty’ and in the descriptive sense referring to specific classes of identity management solutions utilising blockchain-based DPKI. The latter point is also illustrated by the fact that SSI systems are essentially agnostic towards types of entities that can be identified, and can provide a solution for the identification of, say, hardware elements in the Internet of Things systems. In that technical sense, ‘Self-sovereignty’ refers rather to the root of trust in the very specific technical sense (Conway et al. 2019).^20^ This observation also explains that, despite seeming connections with the original blockchain Bitcoin implementation deeply intertwined with libertarian ideas, technical label of SSI systems has a much more neutral normative meaning. It also partially explains why this label is being empathetically embraced by the wide range of actors who could hardly be suspected to be firebrand supporters of crypto-anarchy. Microsoft, IBM, World Bank, and even the US Department of Homeland Security (Funding donor of Evernym Inc.—developer of Sovrin) are just a few such examples.

This rift between technical and normative concepts in itself is not particularly problematic, given that conceptual slippage is a very common occurrence in computer sciences, where concepts are borrowed from the social context, and used in narrow meaning such as ‘trust’ or ‘gossip’. And yet there are instances when concepts borrowed from the social domains lose their original meaning in the technological context, but then get transferred back to the social context carrying over new semantics and new normative content, as, for instance, in the case of ‘trust’ (Ishmaev 2018). China’s Social Credit System provides an illustration of such a feedback cycle where the concept of ‘trust’ becomes applied in social systems, just as it is used in a field of cybersecurity. Here trust is just an operationalised parameter in the system used to discriminate between ‘trusted’ and ‘untrusted’ entities in order to distribute access to the resources of the system (Engelmann et al. 2019).

It is a vivid and uncanny illustration of Chaum’s (1985) prophetic warnings that security principles of hierarchal computer systems can easily become blueprints for social relations. While it would be too pessimistic to expect the complete depreciation of the moral semantics of ‘self sovereignty’, this is not an impossible outcome. Far too often, highly moralised concepts become co-opted by various actors operationalising them in pragmatic way as legitimising claims to sovereign power. Given these observations, it is possible to say that normative assumptions do not disappear from the process of technological development, but rather become implicit and even obscured. Thus, it becomes a crucial task to locate a valid moral foundation to the normative claims of self-sovereignty. A failure to do so carries risks that moral promises of SSI solutions will be distorted and ultimately unfulfilled in the highly adversarial environment, which is the arms race for the control of one’s identity.

## Moral foundations of sovereign rights

A starting point in the quest to locate such a moral foundation is to look at how those components are defined by the proponents of SSI. Possibly the most explicit formulation of principles of sovereignty in the context of SSI technologies can be attributed to Allen (2017), who should also be credited with the popularisation of the term itself. He formulates normative principles that discern decentralised digital identity management systems from other centralised and federated schemes. Some of the principles suggested by Allen are more concrete, such as the necessity of open-source software for the implementation of SSI systems, together with calls for the standardisation of digital identity formats allowing interoperability and portability. Other principles are more general and refer less to concrete technical aspects but rather to governance aspects of SSI systems, such as the use of decentralised databases, the absence of gatekeeping authorities and adherence to minimisation of data disclosures. And finally, principles that can be considered as explicitly ethical ones—the importance of informed consent, the right to be forgotten and control over choice of identity verifiers for system users.

But it is the very first of Allen’s principles—labeled ‘Existence—’ that could be considered a key broad motivational principle with strong moral and political connotations:

Users must have an independent existence. Any self-sovereign identity is ultimately based on the ineffable “I” that’s at the heart of identity. It can never exist wholly in digital form. This must be the kernel of self that is upheld and supported. A self- sovereign identity simply makes public and accessible some limited aspects of the “I” that already exists.

One fruitful interpretation suggests that this statement about identity independence aims to target this very loaded set of moral and political aspirations regarding individual rights to self-determination, at least in part connected with identity and conceptions of self. Such interpretation in itself, of course, hardly amounts to satisfactory clarification, considering the battled status of definitions for these rights in legal and moral philosophy. Yet, it highlights the strong moral aspirations present in the ideas of ‘individual sovereignty’. Another rather insightful suggestion on the moral interpretation of sovereignty of identity is suggested by Loffreto (2012) and Marlinspike (2014), who argue on the necessity of recognition of an individual human right to possess data relational to ones individual identity, (credited by Allen as a sources of inspiration for his work on the principles on self-sovereignty). This argumentation demonstrates a strong libertarian leaning in the critique of exclusive rights possessed by the government to issues and assigning identities to its citizens. While this position is not developed in sufficient details, it can be taken as a certain illustrative point, providing direction for further investigation.

In the broader family of blockchain solutions, normative claims on the moral value of self-sovereignty seem to take a prominent place as well. As Reijers et al. (2016) argue, self-sovereignty in this context can be understood as a guiding governance principle in the design of original blockchain protocols, reminiscent of arguments found in some of the traditions of politico-philosophical theorising. They suggest that ‘self-sovereignty’, can be understood as a principle of the decentralisation of power—very much in the vein of Rousseau’s ideas of decentralised governance. And rather counterintuitively, they reveal some components of the social contract theory suggested by Hobbes, drawing parallels with the assumptions on the self-serving motivations of the participants. The latter comparison is particularly interesting, given that Reijers et al. (2016) demonstrate an uncanny resemblance between Hobbsean delegation of individual rights to abstract power of ‘Leviathan’, as a stabilising mechanism of interaction between humans driven by selfish interests, and rules of blockchain protocol stabilising pre-given system of human interactions (property, insurance system) as ‘Techno-Leviathan’.^21^ It seems then that it might be fruitful to dig a bit deeper into the moral-theoretical arguments on the sovereignty and distribution of power.

Interestingly enough, in the context of SSI technologies, the argumentation on the individual rights to autonomy shows a strong parallel with a Lockean classic liberal critique on the sovereignty, government, and sources of human rights. In the ‘Two Treatises of Government—’ work foundational to the modern theory of human rights—Locke targets the idea of sovereign monarchy as a foundation of state and citizenship, juxtaposing to it the normative concept of natural rights (Locke et al. 2003 [1823]).^22^ Indeed, the invention of the modern passport as an identification system derives directly from the idea of a sovereign nation state, an exclusive right of a national government to provide and demand identities, circumscribed by the scope of the territorial sovereignty (Lloyd 2008). In that sense the right of a national state to issue and demand identification for everyone within its territorial scope is reminiscent of an absolute sovereign right, a self-legitimising fact that requires no external justification.

From that perspective, the call to reconsider the source of this right aims to reframe the procedure of an identification not as an obligation or duty of citizens to be identified derived from the sovereign right of a state, but as a natural right of an individual to be represented via mediating role of institutions of identity. This argument does seem to fall into a broader Lockean argumentation on the foundational status of natural human rights as the source of moral justification for the functions of the state and civil government (Locke et al. 2003 [1823]). This historic parallel also illustrates another observation, highlighted by the emerging new domains of functional sovereignty—that competing calls to reconsider and redefine sovereignty historically coincided with moments of significant social transformations (Kalmo and Skinner 2010).

To challenge claims on the legitimate sources of sovereignty in the vein of Lockean investigation on the sources of rights and powers, it is not enough to point out the contradictory nature of claims for sovereignty in the vein of empirical analysis highlighted in the previous parts of this paper. It is also necessary to locate the moral foundations of competing claims to sovereignty suggested by the proponents of the right for ‘self sovereign’ identity. This deeper moral theoretical aspect of the aforementioned socio-technical transformations can be found in the debates on the changing status of informational privacy and its intertwinement with the moral issues of personal identity formation. Floridi (2006) suggests such an argument, based on the strong ontological interpretation of personal identity understood in informational terms, where an individual is not just represented by one’s personal information but effectively constituted by an information about oneself. From that perspective, the unique dynamic status of personal identity defines a moral content of informational privacy as a matter of construction of one’s own informational identity. An individual’s freedom to mould one’s identity, the freedom to build a different and possibly better self, goes against the artificial ‘mummification’ of identity represented in records and profiles, which takes the power to construct one’s identity away from an individual.

Shoemaker (2010) arguing against such strong ontological interpretation of informational identity, nevertheless also suggests that the right to informational privacy is also a right to control or manage the presentation of one’s self-identity: a right to manage certain public construals of one’s self-identity, or at least to have a say in determining how one’s identity is interpreted by others. This right, suggests Shoemaker, constitutes a moral objection to data mining and subsequent profiling that effectuates construals of an individual’s identity without his or her input in this process. Manders-Huits and van den Hoven (2008) suggest a somewhat different line of argumentation for informational right to privacy that is directly derived from the principles of moral autonomy, epistemic modesty and respect for the persons. The right to moral autonomy as a precondition for freedom to develop and protect one’s identity provides capacity to shape our own moral biographies, to evaluate and identify with one’s own moral choices, without pressure or inference from others. The fixation of one’s moral identity by others, constrained in the form of database records or identity management systems fails to appreciate the epistemic asymmetry between knowledge by description and first-person knowledge of one’s identity. While the former fixes only facts of biography, the latter is deeply intertwined with one’s thoughts, emotions, aspirations and higher-order evaluations. Respect for privacy of persons from that perspective represents acknowledgement for an epistemic modesty in explicit or implicit claims to know who someone is.

Therefore, argue Manders-Huits and van den Hoven, even when it is impossible to leave it completely to individuals to design their own identities in identity management systems, they have a right to authorise and correct when and where it is appropriate, to avoid a nominalisation of identity, and avoid its reduction to a set of externally imposed identifiers. As different first party and functional third party perspective may be, we should not forget that practical fragments of identity—identifiers, serve as building blocks and tools for the more complex ‘own’ person’s identity. And there are always risks of moral failure when such new tools are introduced. Representation of this aspect of persons is exactly what is missing when personal data is piled up in databases and personal identity become nominalised in administrative procedures (Manders-Huits 2010). This moral failure takes a different dimension when one’s identity is not just nominalised, but also evaluated in the normative framework externally and authoritarianly imposed on the bearer of an identity (Ishmaev and Stokkink 2020).

There are then compelling reasons to consider the right to be a ‘self-sovereign’ source of power to construe one’s own identity. Not just a right for the choice of attributes relevant for the presentation of one’s own identity to others, but also a right not to have one’s identity be permanently fixated in the externally imposed normative framework. The foundation of this right can be traced back to Lockean arguments on the limits of powers and rights in a free society. While these arguments belong to their own historical context, in which Locke is occupied with the question of religious tolerance, these very issues are still foundational in the context of contemporary liberal society as well, as can be seen from the history of identity politics in the twentieth century (Sen 2007). In ‘A Letter Concerning Toleration’ Locke observes that moral actions lie both in the jurisdiction of the “magistrate and conscience” (Locke et al. 2003 [1823]). However, the limit of the civil government, Locke argues, stops in the domain where “one man does not violate the right of another, by his erroneous opinions… nor is his perdition any prejudice to another man’s affairs.” (p. 242).

The domain of moral choices concerning one’s own happiness, argues Locke, belongs to the domain of things “that every man ought sincerely to enquire into himself, and by meditation, study, search, and his own endeavour, attain the knowledge of” (p. 229). Here Locke locates the foundational right to make one’s own moral choices and freely identify with these choices “because no man can so far abandon the care for his own salvation as blindly to leave it to the choice of any other” (p. 219). And accordingly, in the matters concerning moral identity and moral choices regarding one’s own well being, civil government has power only to persuade by reason and press with arguments, but not with the penalties. Thus, it can be said that the parallel between Lockean classical liberalism and more recent arguments on the role of identity-formation in the context of informational privacy is more than just an instructive metaphor. In that sense, the moral right to define one’s own identity is a counterclaim to the nominalisation of one’s identity, to its fixation within externally predetermined frame of attributes. But what is even more important it is a right to define value of one’s own identity, to choose the framework for the evaluation of one’s own identity in the sense of a moral autonomy.

And it would be wrong to interpret this right in the vein of a naive atomistic individualism, as a utopian world of fully self-sufficient individuals. Rather, it should be understood as a claim to a degree of freedom; a free space defined by the right to privacy, but also a space free from the externally imposed judgment on one’s own moral choices. As Sen (2007) rightly points out, an identity cannot be seen as something completely unencumbered by the life circumstances of an individual. However, it is crucial that even in the encumbered position one happens to occupy, choice regarding one’s own identity continues to exist. This is then not a claim for the proclamation of individual atomism, but rather a counterclaim to creeping powers of self-proclaimed sovereign entities: a counterclaim against attempts by those entities to legitimise their powers to assign and evaluate humans’ identities in the new domains of emerging socio-technical systems. And what is more important, this claim re-allocates the moral *raison d’etre* for identity management systems. Existence of such systems is not derived from an obligation or duty of individuals to be identified, but from a right of an individual to be represented on ones own terms by mediating mechanisms of institutions and technologies.

## Conclusion

This paper has provided an outline for the moral grounding of claims on the desirability of SSI solutions. Yet somewhat paradoxically, this very set of moral arguments provides a basis for the sceptical arguments on the ‘identity problem’ motivation behind these implementations. Far too often it is unequivocally assumed that the absence of identification mechanism is the problem that needs a solution. Obscuring the fact that the very framing of this question is the problem in itself—problem rooted in a deeper moral issue of persistent identification creep. Furthermore, if the question on the desirability of identification becomes interpreted in the instrumentalist vein—defined by the parameters of a systems’ efficiency rather than needs of individuals interacting with it—it obfuscates and disguises interests of entities expecting to benefit from the advantageous positions in the new ecosystems enabled by SSI.

The ability to issue one’s own digital identity, to choose a list of presented attributes, and even to choose which entities could verify these attributes, does present a significant shift from centralised identity management systems. All those key properties of SSI systems, that can provide the individual bearer of identity with an enhanced degree of freedom for self-presentation. Furthermore, the capacity to share only those identity attributes that are relevant to given interactions shifts the distribution of power in favour of an identity owner. From that perspective, SSI solutions can claim a valid moral argumentation on the desirability of such systems as compared to the centralised identity management systems. These technical elements in themselves, however, do not guarantee the preservation of morally desirable properties in SSI systems implemented on a scale. As Manders-Huits (2010) points out, the very structure of identity management systems promotes a presupposed, nominal notion of identity, resulting in moral tensions between the system logic and reflexive identification of individual. There is an apprehension then, that such tensions will only sharpen with the further depreciation of moral semantics in ‘self-sovereignty’.

It is also important to appreciate that despite what is argued, in themselves technical elements of SSI solutions do not present a ‘paradigm shift’ (Wagner et al. 2018). In their current form, SSI systems do not challenge the general paradigm of socio-technical systems whose cornerstone design principles are identification-based trust and access control. This observation highlights the risk of a mission creep inherent to identity solutions. The desirable task of translating existing credentials systems into privacy preserving digital formats, can morph into the search for new contexts of application for the existing solution.^23^ This possibility can be illustrated by the proposals suggesting that a cell provider can verify the location of an individual in a deterministic way in the SSI system (Sovrin 2018). It may sound like an eccentric application at a first glance, but once we consider the existing practices of insurance providers installing tracking devices in cars in return for discounts, or court cases using data from ‘wearables’, this identification creep takes on a distinctively dystopian flavour. Which may lead to the normalisation of new standards for cryptographically verified data in the scenarios where individuals previously were not expected to poses and provide such data at all.

This brings about another speculative component of SSI proposals—a promise of a sufficiently decentralised ‘web of trust’ based on a free-market ecosystem of competing verifiers. Such competition would enable individual users to choose between different providers of such services, thus taking away the power from verifiers to dictated standards of identification. The immediate apprehension here is that even with the permissionless blockchain protocols, the achievement of a meaningful decentralisation is a notoriously difficult task.^24^ It is also important to appreciate that the proposed free-market mechanisms aimed to achieve promised decentralisation are not morally unproblematic in themselves. This apprehension becomes clear once we consider proposals on the global marketplaces for credentials and ‘ethical’ markets for customers’ data (Acxiom 2017; Sovrin 2018; Wagner et al. 2018). Such proposals essentially run into the fallacy that free-market mechanisms can bring about morally desirable outcomes—assumption largely construed on the idealised representation of the rationality of such markets. What these assumptions, however, largely ignore is a risk that such market mechanisms would rather fit into the structures of existing private data markets, replicating and even exaggerating moral risks associated with the private data propertization (Ishmaev 2019).

True enough, cryptographic solutions such as pairwise identifiers, can present a barrier against adversarial profiling, preventing third parties from the aggregation of profiles on DID owners. Yet, there is no such guaranteed technical solution that could prevent uses of a single identity or a limited set of identifiers by individuals in SSI systems. And this is not merely a problem of technical design, or education of users as in the case of, say, reuse of the same wallet address by Bitcoin users (Wagner et al. 2018). This is also a critical issue of establishing standards on the desirability of minimised, plural identities: disposable ‘personas’, pseudonyms, or even anonymous digital credentials. And the biggest non-technical challenge to the facilitation of ‘self-sovereignty’ in SSI systems in a strong sense, is a debunking of claims on the absolute moral desirability of a strong singular identity. Otherwise, the very same infrastructures enabling SSIs will be re-purposed to facilitate aggregation of profiles and scores. Here, interoperability and standardisation could play a treacherous role, facilitating the emergence of standardised reputation systems, with normative framework of identity evaluation externally imposed on individuals.

It is also not enough to merely claim moral motivation for the development of SSI systems derived from the right of an individual to be represented via the mediating role of socio-technical solutions in the Lockean vein of thinking. We should also keep in mind that with the growing dependence of individuals on technical infrastructures, even systems designed as opt-in can quickly become de-facto necessities with the wider adoption and network effects. And any proposed ‘identity solution’ scenario should always be evaluated against with the possibility of an alternative solution that does not require any persistent identity at all. Thus, there is a task for the development of a moral-theoretical framework bringing scrutiny to the desirability of identification solutions. Only doing so we can ensure that SSI systems contribute to the realisation of a ‘self-sovereignty’ ideals rather than to the emergence of a Hobbsean ‘Techno-Leviathan’.

## Footnotes

1 The Cryptography Mailing List. Full context of citation is a response in the mailing list: “You will not find a solution to political problem in cryptography”—“Yes, but we can win a major battle in the arms race and gain a new territory of freedom for several years”. Available at: https://nakamotostudies.org/emails/re-bitcoin-p2p-e-cash-paper-3/.

2 Bitcoin was influenced by a long line of successive projects, attempting to implement cryptographically based digital means of exchange including Chaum’s ‘Digicash’. For historical overview of these implementations see Narayanan et al. (2016).

3 Term 'balkanization of the Internet’ is used in different discourses, and can refer to a wide range of issues. Here, it refers to the increasing legislative and technological measure of national governments, aimed to ensure control over certain segments of the Internet. These measures include localisation of data in rest and in transit within physical boundaries of nationals state, censorships measures targeting national segments of the Internet, and measure aimed to ensure compliance of service providers with local regulations. For an extended critique of this issue see. Hon et al. (2016).

4 Widely publicised legal battle between Apple and FBI, regarding access to data encrypted on Apple hardware, is just one of such examples (Zetter 2016). Or concessions that Apple made to the Chinese government regarding the storage of Chinese Apple users’ data.

5 A recent controversy surrounding the development of a smart-city project in Toronto provides a glimpse of such future contradictions (Canon, 2018).

6 ‘Facebook connect’ and ‘Google sign-up’ respectively are sets of Application Programming Interfaces (API), that can be implemented by third party web-services (websites, apps, etc.) to let their visitors authenticate themselves using Facebook or Google identities.

7 As Dixon observes, by the time Aadhaar system passed the barrier of one billion enrolments in 2016, Indian government still has no passed national data protection and privacy legislation. And more astonishingly by the time of passing of ‘Aadhaar act’ regulation the system has been in place effectively for 7 years.

8 Aadhaar provider UIDAI tries to mitigate some of the privacy concerns with the introduction of virtual IDs that can be used for verification purposes without revealing unique identifying number, to prevent third party identity correlation. It has been argued, however, that this retrofitted mechanism does not address more fundamental issue of data abuse enabled by centralized silos for biometric data (Banerjee and Sharma 2019).

9 Another vivid and disturbing example is an educational program for schoolchildren designed and financed by Google with a stated aim to tech children ‘the fundamentals of digital citizenship’, which presents Google to pupils as an impartial and trustworthy entity (Singer & Maheshwari 2018).

10 Truly bizarre examples can be found in different consumer applications of solutions for the ‘identity assessment’, now offering even algorithmic assessments for the trustworthiness of baby seaters, on the basis of social network data (Harwell 2018).

11 Microsoft, Accenture and Avanade, are partners of ID2020 alliance, collaborating on the development of blockchain based ‘self- sovereign’ identity solutions. https://blogs.microsoft.com/blog/2018/01/22/partnering-for-a-path-to-digital-identity/.

12 Strictly speaking not all these projects aim to provide full solutions, but the list is representative. See: https://github.com/peacekeeper/blockchain-identity.

13 See https://www.uport.me/.

14 See https://sovrin.org/.

15 It should be noted that these standards are not blockchain specific, however, it is assumed that practical implementations currently are most feasible on the basis of blockchain technologies.

16 In certain implementations, such as Evernym, DID can be written directly to the ledger as a public DID, or stored off-ledger and shared privately between identity holders. See: https://www.evernym.com/wp-content/uploads/2017/07/What-Goes-On-The-Ledger.pdf.

17 It needs to be noted that uPort can be compatible with W3C specific DID standard. See for instance: https://github.com/uport-project/secp256k1-did-resolver.

18 This is also why prototypic blockchain applications are called ‘permissionless’, since there are no restriction on who can join the network and use its resources. Factual decentralisation, of course, is a separate issue considering that concentration of hashing power in the hands of entities controlling superior economic resources is a very real possibility. At the moment this balance is very fragile to say the least, but seem to improve at the moment of writing of this paper. See: https://www.blockchain.com/pools.

19 Again, predetermined power relations are not necessarily problematic in themselves. Mere replication of government ID via SSI scheme within the institutionalised legal and ethical frameworks arguably does not necessarily invoke novel moral issues.

20 In general terms the ‘root of trust’ is a source that can always be trusted within a cryptographic system..

21 This parallel, however, breaks apart with the observation that participation in any blockchain application is completely voluntary, and unlike Hobbsean ‘Leviathan’, power of blockchain protocol is not sustained by the constant threat of punishment.

22 Locke in ‘Two Treaties of Government’ juxtaposes the moral claim to individual right and monarchical claims to sovereign power, providing a strong rebuttal of the latter. Granted, here it could be objected that Locke does not propose an idea of individual sovereignty, but that of an individual right. Yet as Baranger argues (2010) despite apparent unlikeness the comparison of these concepts is not unjustified, both concepts in the original sense aim to highlight an individual in the legal sense, and both aim to locate source of right and duties in an individual. In the sense Lockean definition of individual right is actually conceptually closer to the original meaning of individual sovereignty, attributed to monarch as an individual bearer.

23 This became particularly evident in the year of COVID-19 pandemic, with a wide range of identity management system providers racing to repurpose their solutions for ‘immunity certificates’ and ‘vaccination certificates’.

24 Not to mention the issue of highly contestable criteria of a ‘sufficient’ decentralization.
